# National Early Warning Score 2 and laboratory predictors correlate with clinical deterioration in hospitalized patients with COVID-19

**DOI:** 10.2217/bmm-2021-0061

**Published:** 2021-07-21

**Authors:** Gulsah Tuncer, Serkan Surme, Osman F Bayramlar, Hatice K Karanalbant, Betul Copur, Meltem Yazla, Esra Zerdali, Inci Y Nakir, Ayse RK Cinar, Ahmet Buyukyazgan, Hatice Balli, Yesim Kurekci, Serap Simsek-Yavuz, Mehmet M Sonmez, Gonul Sengoz, Filiz Pehlivanoglu

**Affiliations:** 1 Department of Infectious Diseases & Clinical Microbiology, Haseki Training & Research Hospital, Istanbul, Turkey; 2 Department of Public Health, Bakirkoy District Health Directorate, Istanbul, Turkey; 3 Department of Infectious Diseases & Clinical Microbiology, Istanbul Faculty of Medicine, Istanbul University, Istanbul, Turkey; 4 Department of Orthopaedic Surgery & Traumatology, Haseki Training & Research Hospital, Istanbul, Turkey

**Keywords:** albumin, COVID-19, in-hospital mortality, neutrophil/lymphocyte ratio, NEWS2, procalcitonin

## Abstract

**Aim:**

We aimed to determine the prognostic values of the National Early Warning Score 2 (NEWS2) and laboratory parameters during the first week of COVID-19.

**Materials & methods:**

All adult patients who were hospitalized for confirmed COVID-19 between 11 March and 11 May 2020 were retrospectively included.

**Results:**

Overall, 611 patients were included. Our results showed that NEWS2, procalcitonin, neutrophil/lymphocyte ratio and albumin at D0, D3, D5 and D7 were the best predictors for clinical deterioration defined as a composite of ICU admission during hospitalization or in-hospital death. Procalcitonin had the highest odds ratio for clinical deterioration on all days.

**Conclusion:**

This study provides a list of several laboratory parameters correlated with NEWS2 and potential predictors for clinical deterioration in patients with COVID-19.

## Lay abstract

The COVID-19 pandemic is a grueling problem worldwide. There is a lack of knowledge about the predictive value of National Early Warning Score 2 (NEWS2) for severe COVID-19 illness. We analyzed the prognostic value of NEWS2 and laboratory parameters during the clinical course of COVID-19. This study provides a list of several laboratory parameters correlated with NEWS2 and potential predictors for intensive care unit admission during hospitalization or in-hospital death.

The COVID-19 pandemic due to the SARS-CoV-2 virus causes high rates of mortality, morbidity, longer duration of hospitalization and increased need for intensive care unit (ICU) admission [1]. Improving critical care patient flow is crucial for high-quality care in severe cases. Therefore, we need to predict clinical deterioration in patients with COVID-19, in order to hospitalize the patients and admit to ICU, when necessary. The National Institute for Health and Care Excellence recommend the National Early Warning Score 2 (NEWS2) to predict the risk for clinical deterioration in patients with COVID-19 [2,3]. NEWS2 is a simple scoring system including physiological parameters and vital signs (respiratory rate, oxygen saturation, systolic blood pressure, heart rate, level of consciousness, body temperature and supplemental oxygen dependency) used to predict the risk for acute deterioration including sepsis [4,5]. An increasing number of studies have assessed the physiological parameters, vital signs and some scoring systems for severe COVID-19 illness [6,7]. However, there is a lack of knowledge about the predictive value of NEWS2, despite some studies focus on NEWS2 and related scores [3].

To date limited data exists on the NEWS2 and laboratory parameters in patients with COVID-19. In this study, we aimed to determine prognostic value of NEWS2 and laboratory parameters during the clinical course of COVID-19. Additionally, the correlation between NEWS2 and laboratory parameters at admission (D0), day 3 (D3), day 5 (D5) and day 7 (D7) were evaluated. To our knowledge, this is the first study evaluating both NEWS2 and laboratory parameters on the clinical course of COVID-19 in Turkey.

## Materials & methods

### Study design & patients

In this retrospective and single-center study, all adult patients (≥18 years old) who were hospitalized for a laboratory confirmed COVID-19, between 11 March and 11 May 2020 were included. SARS-CoV-2 testing was performed using real-time reverse transcription-PCR of samples collected by nasopharyngeal and/or oropharyngeal swabs.

Patients with COVID-19 requiring hospitalization were included in the study. Outpatients and asymptomatic patients were excluded. Also we excluded patients if oropharyngeal or nasopharyngeal swab samples were repeatedly negative for SARS-CoV-2 by reverse transcription-PCR. Our primary outcome was the occurrence of clinical deterioration defined as a composite of ICU admission during hospitalization or in-hospital death.

The criteria for ICU transfer in our hospital were the following parameters (at least one or more); dyspnea and respiratory distress; respiratory rate ≥30/min; oxygen saturation <90% or partial oxygen pressure <60 mmHg despite oxygen support (≥5 l/min); septic shock and/or multiple organ dysfunction.

### Data collection

Epidemiological and demographic characteristics, clinical, laboratory, radiological findings and outcomes were collected from medical records. Vital signs including respiratory rate, peripheral capillary oxygen saturation, heart rate, blood pressure, body temperature and consciousness (Glasgow coma scale) were recorded. Laboratory parameters including albumin, C-reactive protein (CRP), procalcitonin, hemoglobin, hematocrit, neutrophil count, lymphocyte count, platelet count, neutrophil/lymphocyte ratio (NLR), platelet/lymphocyte ratio (PLR), urea, ferritin, albumin, fibrinogen, D-dimer, aspartate aminotransferase (AST) and alanin aminotransferase at D0, D3, D5 and D7 were included. The NEWS2 score was calculated at D0, D3, D5 and D7. These parameters were obtained at the same time.

### Statistical analysis

Quantitative variables are expressed as mean and standard deviation when they contain continuous and normal distributed data. When the data were not distributed normally, median and interquartile range (IQR) were used. When they contained categorical data, they were expressed as a percentage (%) and frequency (n). Comparison of qualitative variables was performed by Pearson’s Chi-square test. The normal distribution questioning the necessity of using the parametric test was examined by Kolmogrov–Smirnov, Shapiro–Wilk, Kurtosis–Skewness tests and box plot distribution. When normally distributed data could not be determined, nonparametric tests and spearman correlation were used. Kruskal–Wallis test was used for the analysis of continuous and more than two independent nonparametric groups (Bonferroni correction was used when necessary) and Mann–Whitney test was used for *post hoc* analysis. To evaluate the factors in prognosis which were admission ICU and in-hospital death, univariate logistic regression analysis was performed. Afterward, these dependent groups were handled one by one, receiver operating characteristic (ROC) curves were drawn and cut-off values, sensitivity and specificity and area under the curve (AUC) were demonstrated. To predict clinical deterioration, the prognostic accuracy of NEWS2 and laboratory parameters at D0, D3, D5 and D7 was evaluated by ROC analyses.

- NEWS2 and laboratory parameters at D0;
- NEWS2 and laboratory parameters at D3 after excluding from the analysis patients with clinical deterioration within the first 3 days of hospitalization;
- NEWS2 and laboratory parameters at D5 after excluding from the analysis patients with clinical deterioration within the first 5 days of hospitalization;
- NEWS2 and laboratory parameters at D7 after excluding from the analysis patients with clinical deterioration within the first 7 days of hospitalization.

Additionally, the association of the parameters at admission with the clinical deterioration was evaluated to predict the 3-day, 5-day and 7-day end points by ROC analyses.

The results were evaluated in 95% CI and statistical significance level was defined as p < 0.05. The analyzes were performed using IBM SPSS-21 (Statistical Package for Social Sciences, IL, USA).

## Results

### General characteristics

Overall, 611 patients were included. Of whom, 329 (53.8%) were male, the mean age was 52.53 ± 15.07 years. Seventy-three patients (11.9%) were admitted to the ICU. In-hospital death occurred in 46 (7.5%) patients. Among 73 patients (11.9%) admitted to the ICU, 40 patients (54.8%) died during hospitalization. Clinical deterioration was observed in 79 patients (12.9%) during hospitalization, 36 (5.9%), during the first 3 days, 54 (8.8%) during the first 5 days and 62 (10.1%) during the first week of hospitalization. NEWS2 was calculated at D0, D3, D5 and D7 of hospitalization. Patients were stratified into three risk groups: low risk from zero to four; medium risk from five to six and high risk above seven. Of 611 patients, 505 (82.7%) at D0, 411 (91.9%) at D3, 375 (92.2%) at D5, 284 (93.8%) at D7 had a NEWS2 score <7. The median length of hospital stay was 8.9 days and 332 patients (54.3%) who did not have fever and did not need oxygen in the last 48–72 h and meet the criteria for home monitoring were discharged within the first 7 days. Demographic characteristics of hospitalized patients with COVID-19 are available in Supplementary Table 1.

### ICU admission or in-hospital mortality

The parameters associated with admission ICU and in-hospital death at D0, D3, D5 and D7 were NEWS2, lymphocyte count, neutrophil count, platelet count, NLR, PLR, CRP, procalcitonin, D-dimer, troponin, AST, urea, lactate dehydrogenase (LDH) and albumin. The median and IQR values of the laboratory parameters and NEWS2 are represented in Table 1.

**Table 1.** Median and interquartile range values of the parameters.

Parameters	D0		D3		D5		D7	
	Prognosis		Prognosis		Prognosis		Prognosis	
NEWS2	Poor	Good	Poor	Good	Poor	Good	Poor	Good
– IQR	4	3	3	2	3	3	4	2
– Median	6	4	5	3	6	3	7	3
– p-value	0.001		0.001		0.001		0.001	
Leukocyte count	Poor	Good	Poor	Good	Poor	Good	Poor	Good
– IQR	4020	2990	6745	2628	6383	2390	4223	2200
– Median	6075	5930	7905	5800	7910	5880	7620	6220
– p-value	0.26		0.001		0.01		0.04	
Lymphocyte count	Poor	Good	Poor	Good	Poor	Good	Poor	Good
– IQR	538	810	508	750	295	793	786	1600
– Median	1030	1390	805	1420	710	1505	685	1470
– p-value	0.001		0.001		0.001		0.001	
Neutrophil count	Poor	Good	Poor	Good	Poor	Good	Poor	Good
– IQR	4013	2410	5830	2038	6750	1910	3958	1810
– Median	4645	3710	6565	3505	6520	3680	6475	3800
– p-value	0.001		0.001		0.001		0.001	
Platelet count × 10^3^	Poor	Good	Poor	Good	Poor	Good	Poor	Good
– IQR	83	82.75	132.5	120.75	73	163.5	74.5	160
– Median	175	197	200.5	237	206.5	281	229.5	329
– p-value	0.04		0.01		0.001		0.01	
NLR	Poor	Good	Poor	Good	Poor	Good	Poor	Good
– IQR	4.54	2.11	8.28	1.84	7.36	1.86	10.65	1.64
– Median	4.83	2.7	6.6	2.35	8.5	2.29	7.77	2.6
– p-value	0.001		0.001		0.001		0.001	
PLR	Poor	Good	Poor	Good	Poor	Good	Poor	Good
– IQR	129.8	78.2	173.1	101.6	154.9	119.8	280.8	135.2
– Median	180.3	141.5	250.8	162	278.3	180.2	341.3	223.3
– p-value	0.001		0.001		0.001		0.01	
CRP	Poor	Good	Poor	Good	Poor	Good	Poor	Good
– IQR	99.8	65	139	60	115.3	50.3	139.6	48.6
– Median	110.5	33.1	127	29.8	183	24	142.5	19
– p-value	0.001		0.001		0.001		0.001	
**Procalcitonin**	**Poor**	**Good**	**Poor**	**Good**	**Poor**	**Good**	**Poor**	**Good**
– IQR	0.64	0.05	0.79	0.05	1.08	0.03	0.88	0.05
– Median	0.19	0.04	0.45	0.04	0.58	0.04	0.20	0.05
– p-value	0.001		0.001		0.001		0.01	
D-dimer	Poor	Good	Poor	Good	Poor	Good	Poor	Good
– IQR	1.06	0.64	3.82	0.76	5.97	0.95	4.47	1.11
– Median	0.8	0.63	1.4	0.8	1.4	1	2.7	1.3
– p-value	0.03		0.001		0.01		0.04	
Ferritin	Poor	Good	Poor	Good	Poor	Good	Poor	Good
– IQR	416	218	840	174	4008	241	1631	190
– Median	234	146	583	171	500	203	499	208
– p-value	0.01		0.001		0.01		0.16	
Troponin	Poor	Good	Poor	Good	Poor	Good	Poor	Good
– IQR	39	4.40	29.80	3.40	35.85	2.92	44.90	4.00
– Median	14.6	3.9	12.5	3.2	27.5	3.3	19.1	4
– p-value	0.001		0.001		0.01		0.01	
AST	Poor	Good	Poor	Good	Poor	Good	Poor	Good
– Mean	23	19	41	24	24	21	27	22
– Median	36.5	30	51	31	48	36	46.5	36
– p-value	0.001		0.001		0.001		0.04	
ALT	Poor	Good	Poor	Good	Poor	Good	Poor	Good
– IQR	13	19	34	26	28	32	30	37
– Median	22.5	23	27	26	30.5	32	28.5	35.5
– p-value	0.86		0.13		0.95		0.31	
Creatinine	Poor	Good	Poor	Good	Poor	Good	Poor	Good
– IQR	0.52	0.30	0.80	0.25	1.14	0.25	0.97	0.20
– Median	0.98	0.72	0.86	0.7	0.9	0.68	0.9	0.7
– p-value	0.001		0.001		0.02		0.09	
Urea	Poor	Good	Poor	Good	Poor	Good	Poor	Good
– IQR	30	12	34	12	46	12	51	13
– Median	39.4	27	43.7	25	43.5	24	33	25.8
– p-value	0.001		0.001		0.001		0.01	
LDH	Poor	Good	Poor	Good	Poor	Good	Poor	Good
– IQR	197	114	250	137	151	128	196	138
– Median	336	262	468	272	513	281	485	289
– p-value	0.001		0.001		0.001		0.001	
CPK	Poor	Good	Poor	Good	Poor	Good	Poor	Good
– IQR	210	124	360	80	263	58	457	55
– Median	170	102	181	72	124.5	63	161.5	57
– p-value	0.02		0.001		0.08		0.01	
Albumin	Poor	Good	Poor	Good	Poor	Good	Poor	Good
– IQR	8	5	6	5	7	5	9	4
– Median	33	37	31	36.5	27.5	35	30	34
– p-value	0.001		0.001		0.001		0.001	
Hemoglobin	Poor	Good	Poor	Good	Poor	Good	Poor	Good
– IQR	2.4	2.0	2.5	2.2	2.0	1.9	2.7	2.0
– Median	13	13	11.95	12.6	11.25	12.4	11.7	12.5
– p-value	0.43		0.01		0.001		0.09	
Hematocrit	Poor	Good	Poor	Good	Poor	Good	Poor	Good
– IQR	7.1	5.1	5.7	5.2	4.8	6.0	6.1	5.8
– Median	38.9	39	36.05	37.7	34.3	37.1	35.6	36.7
– p-value	0.24		0.01		0.001		0.16	

ALT: Alanine aminotransferase; AST: Aspartate aminotransferase; CPK: Creatine phosphokinase; CRP: C-reactive protein; D0: Admission; D3: Day-3; D5: Day-5; D7: Day-7; IQR: Interquartile range; LDH: Lactate dehydrogenase; NLR: Neutrophil/lymphocyte ratio; PLR: Platelet/lymphocyte ratio.

### Univariate analysis

In univariate analysis, among parameters associated with ICU admission or in-hospital death at D0, D3, D5 and D7, best predictors were NEWS2, procalcitonin, NLR and albumin. Additionally, D-dimer (at D0, D3 and D7) and hemoglobin (at D3 and D5) were valuable predictors in univariate analysis (Table 2).

**Table 2.** Univariate analysis of parameters associated with ICU admission and in-hospital death.

Parameter	p-value	OR	95% CI
NEWS2 at D0	<0.001	1.386	1.255–1.530
NEWS2 at D3	<0.001	1.709	1.463–1.998
NEWS2 at D5	<0.001	1.933	1.564–2.389
NEWS2 at D7	<0.001	2.030	1.560–2.642
NLR at D0	<0.001	1.286	1.190–1.390
NLR at D3	<0.001	1.754	1.522–2.021
NLR at D5	<0.001	1.806	1.495–2.180
NLR at D7	<0.001	1.332	1.168–1.520
Albumin at D0	<0.001	0.782	0.714–0.856
Albumin at D3	<0.001	0.699	0.627–0.779
Albumin at D5	<0.001	0.752	0.664–0.851
Albumin at D7	<0.001	0.679	0.547–0.843
Hemoglobin at D3	0.003	0.779	0.661–0.919
Hemoglobin at D5	<0.001	0.615	0.470–0.804

ALT: Alanine aminotransferase; AST: Aspartate aminotransferase; CPK: Creatine phosphokinase; CRP: C-reactive protein; D0: Admission; D3: Day-3; D5: Day-5; D7: Day-7; LDH: Lactate dehydrogenase; NLR: Neutrophil/lymphocyte ratio; OR: Odds ratio; PLR: Platelet/lymphocyte ratio.

### Correlation

**Figure 1.**
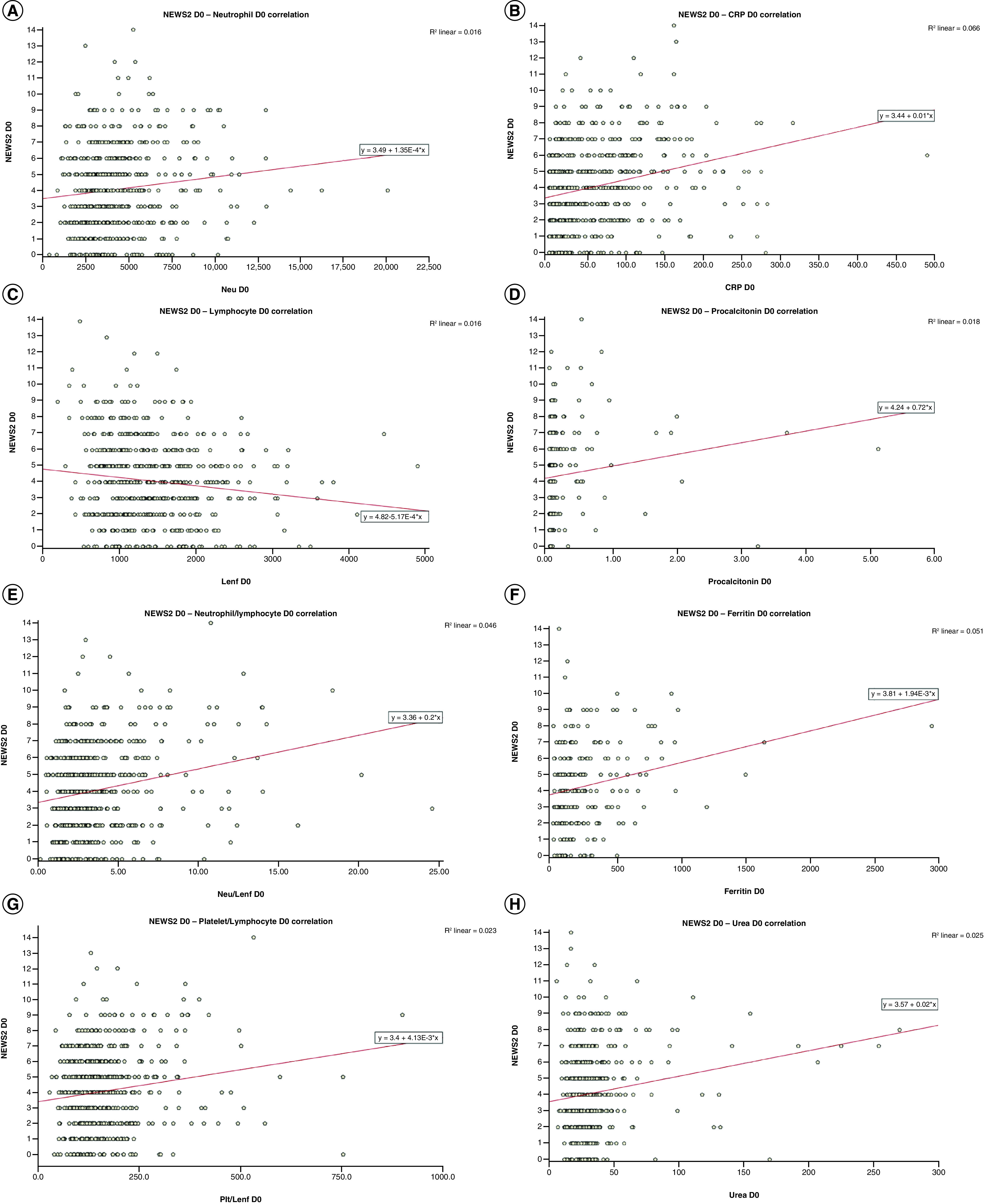
Correlation between National Early Warning Score 2 and lymphocyte count, neutrophil count, neutrophil/lymphocyte ratio, platelet/lymphocyte ratio, C-reactive protein, procalcitonin, ferritin and urea at D0.

**Figure 2.**
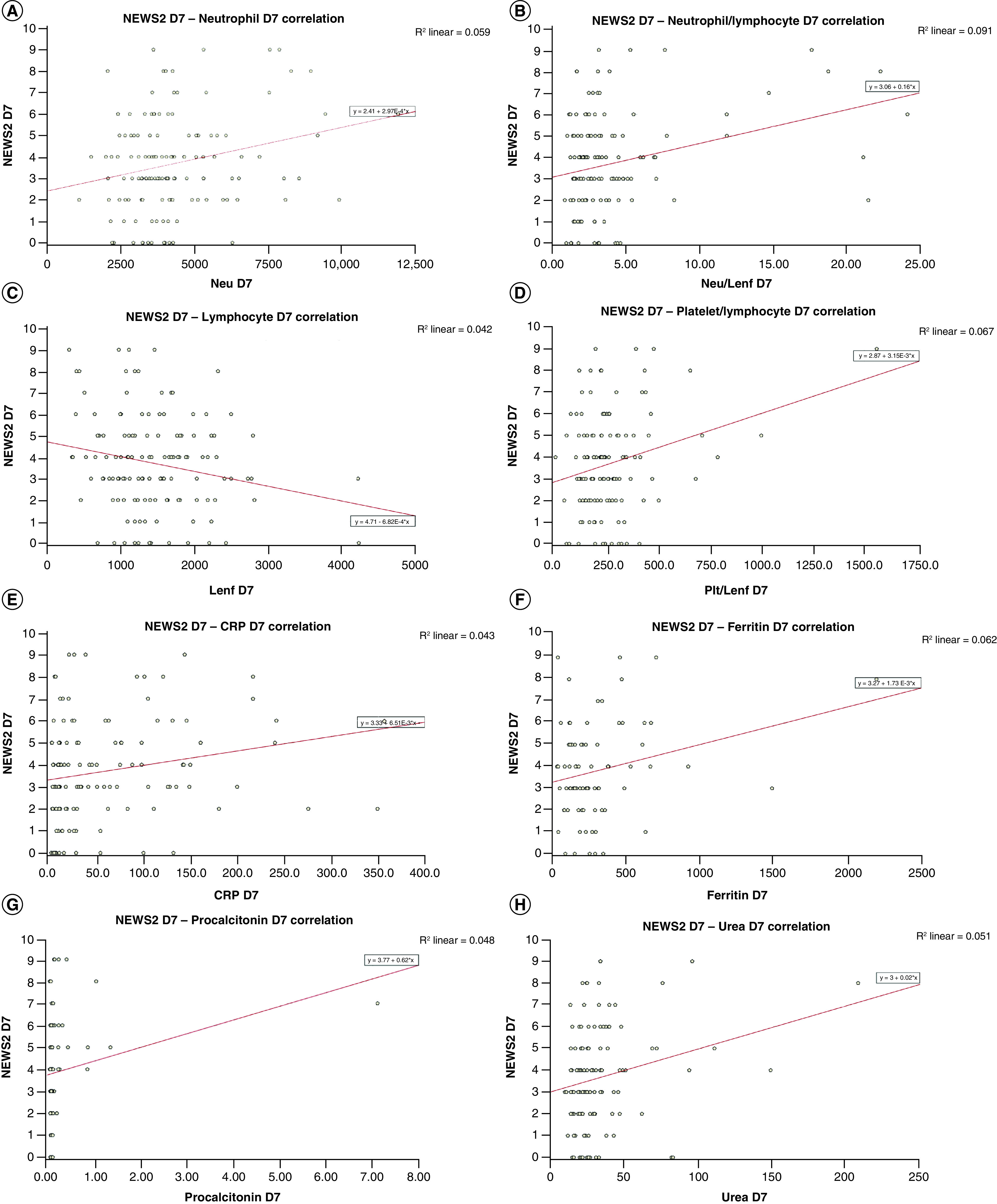
Correlation between National Early Warning Score 2 and lymphocyte count, neutrophil count, neutrophil/lymphocyte ratio, platelet/lymphocyte ratio, C-reactive protein, procalcitonin, ferritin and urea at D7. AUC: Area under the ROC curve; NEWS2:National Early Warning Score 2.

Laboratory parameters correlated with NEWS2 at D0, D3, D5 and D7 were lymphocyte count, neutrophil count, NLR, PLR, CRP, procalcitonin, ferritin and urea (Figures 1 & 2).

### ROC curves

**Figure 3.**
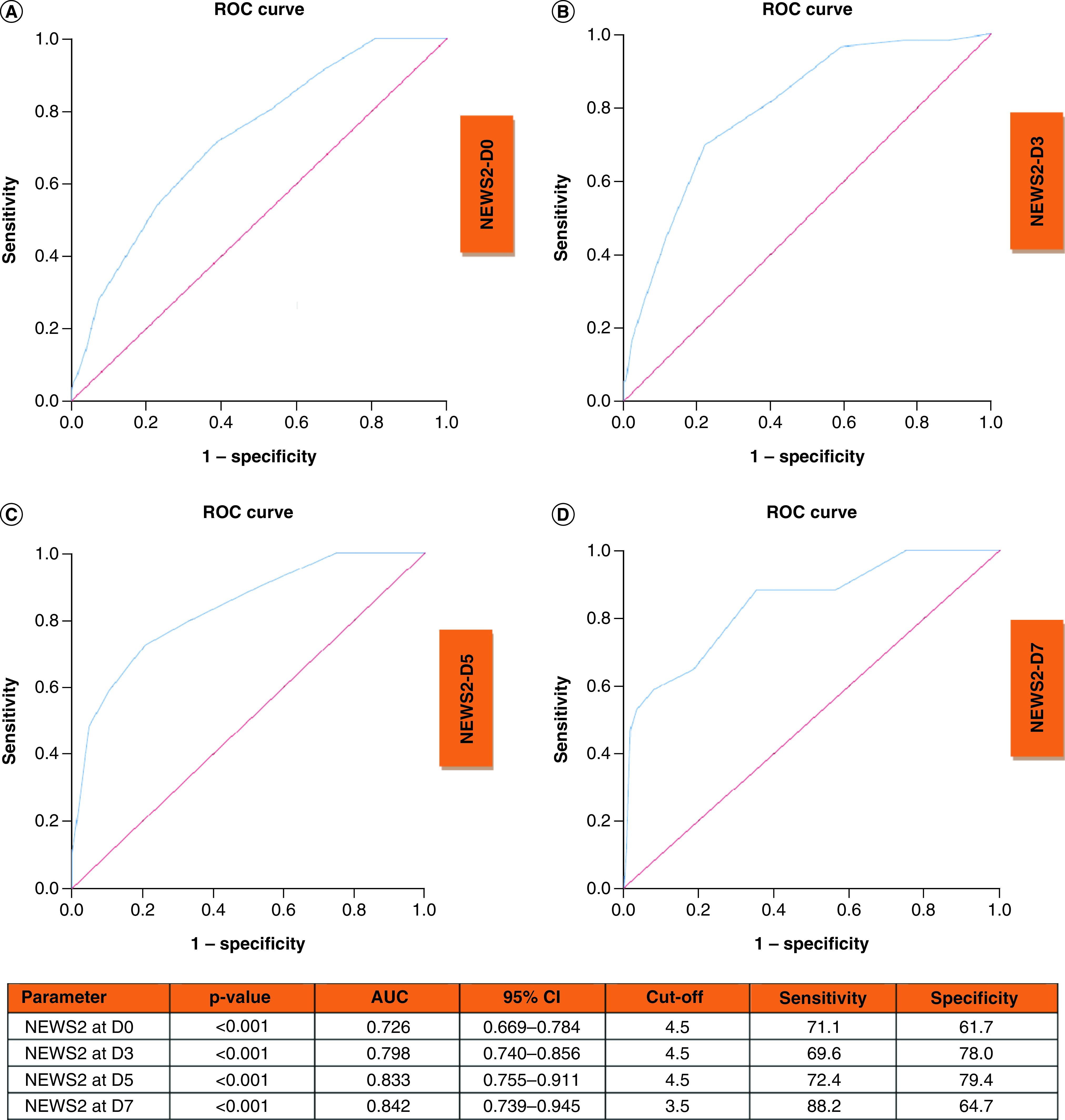
Receiver operating characteristic curves of National Early Warning Score 2 at D0, D3, D5 and D7 to predict clinical deterioration. Receiver operating characteristic curve and performance value for the best cut off for: **(A)** NEWS2 at admission using clinical deterioration. **(B)** NEWS2 at D3 using clinical deterioration. **(C)** NEWS2 at D5 using clinical deterioration. **(D)** NEWS2 at D7 using clinical deterioration. AUC: Area under the ROC curve; D3: Day-3; D5: Day-5; D7: Day-7; ROC: Receiver operating characteristic.

ROC curves of NEWS2 at D0, D3, D5 and D7 to predict clinical deterioration are shown in Figure 3. AUC curves at D0, D3, D5 and D7 were 0.726, 0.798, 0.833 and 0.842, respectively (all p < 0.001).

**Figure 4.**
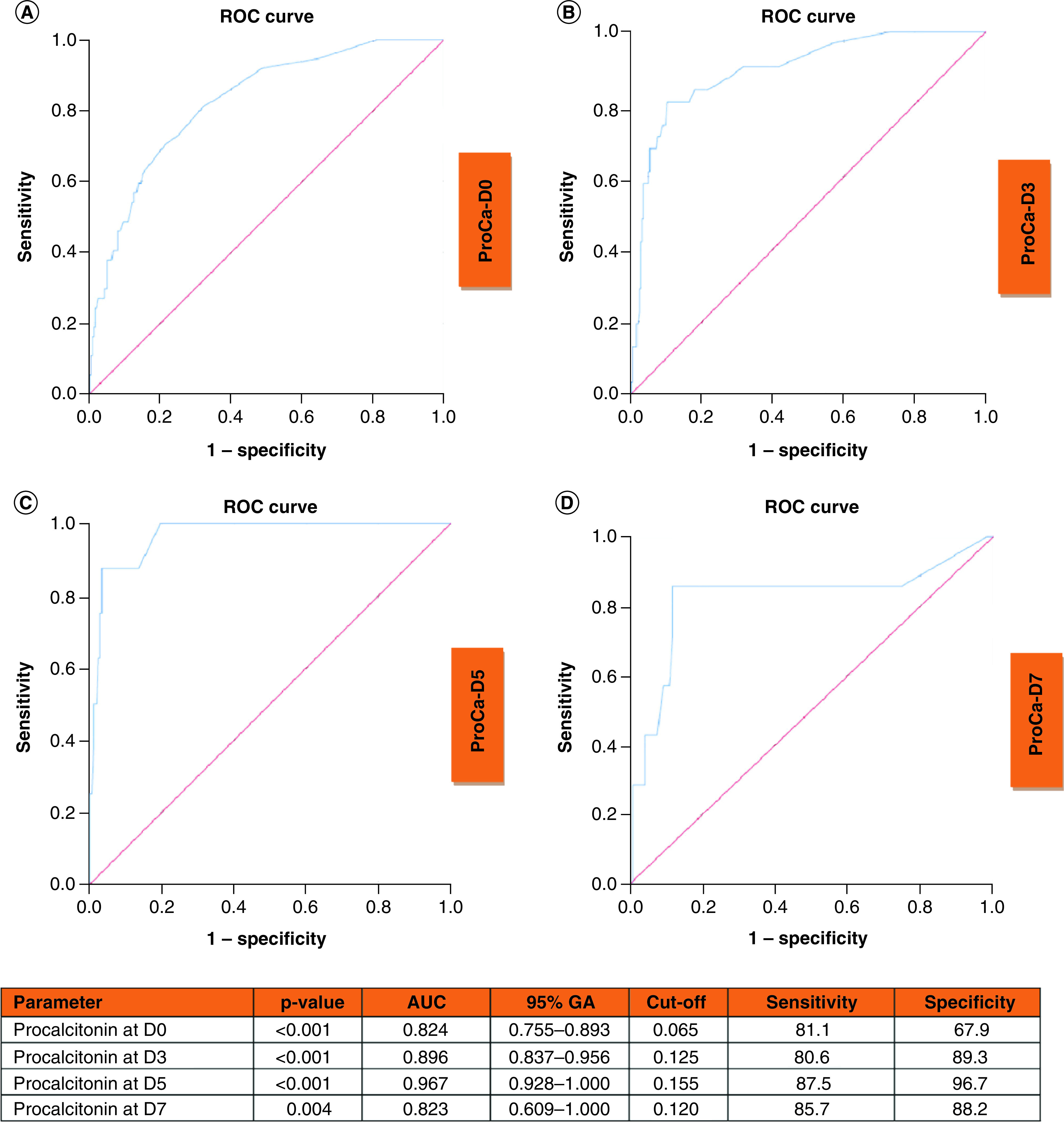
Receiver operating characteristic curves of procalcitonin at D0, D3, D5 and D7 to predict clinical deterioration. Receiver operating characteristic curve and performance value for the best cutoff for: **(A)** Procalcitonin at admission using clinical deterioration. **(B)** Procalcitonin at D3 using clinical deterioration. **(C)** Procalcitonin at D5 using clinical deterioration. **(D)** Procalcitonin at D7 using clinical deterioration. AUC: Area under the ROC curve; D3: Day-3; D5: Day-5; D7: Day-7; ROC: Receiver operating characteristic.

ROC curves of procalcitonin at D0, D3, D5 and D7 to predict clinical deterioration are shown in Figure 4. AUC curves at D0, D3, D5 and D7 were 0.824, 0.896, 0.967 and 0.823, respectively (all p < 0.001; p < 0.001; p < 0.001; p = 0.004, respectively).

**Figure 5.**
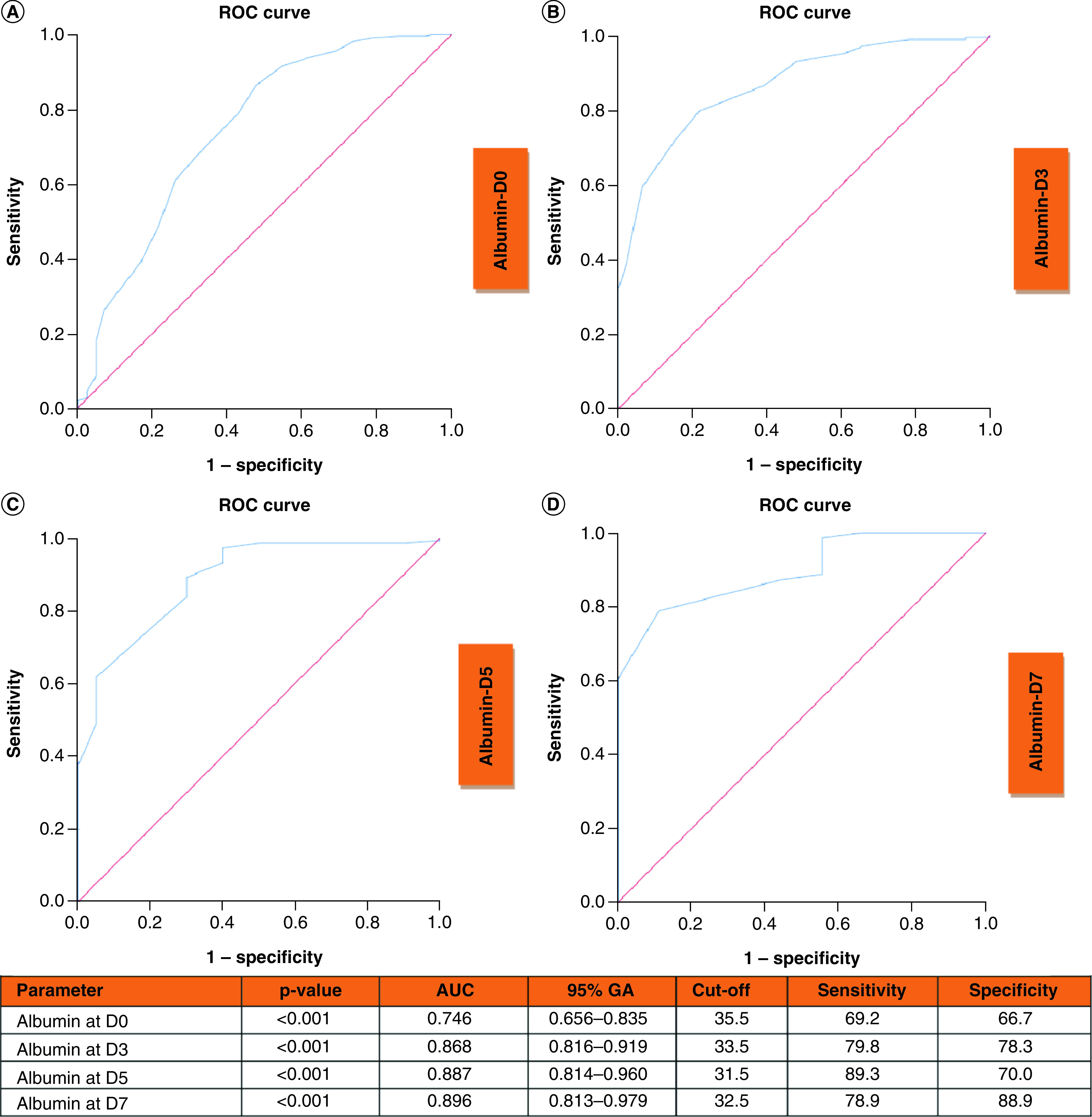
Receiver operating characteristic curves of albumin at D0, D3, D5 and D7 to predict clinical deterioration. Receiver operating characteristic curve and performance value for the best cutoff for: **(A)** Albumin at admission using clinical deterioration. **(B)** Albumin at D3 using clinical deterioration. **(C)** Albumin at D5 using clinical deterioration. **(D)** Albumin at D7 using clinical deterioration. AUC: Area under the ROC curve; D3: Day-3; D5: Day-5; D7: Day-7; NEWS2: National Early Warning Score 2; ROC: Receiver operating characteristic.

ROC curves of albumin at D0, D3, D5 and D7 to predict clinical deterioration are shown in Figure 5. AUC curves at D0, D3, D5 and D7 were 0.746, 0.868, 0.887 and 0.896, respectively (all p < 0.001).

**Figure 6.**
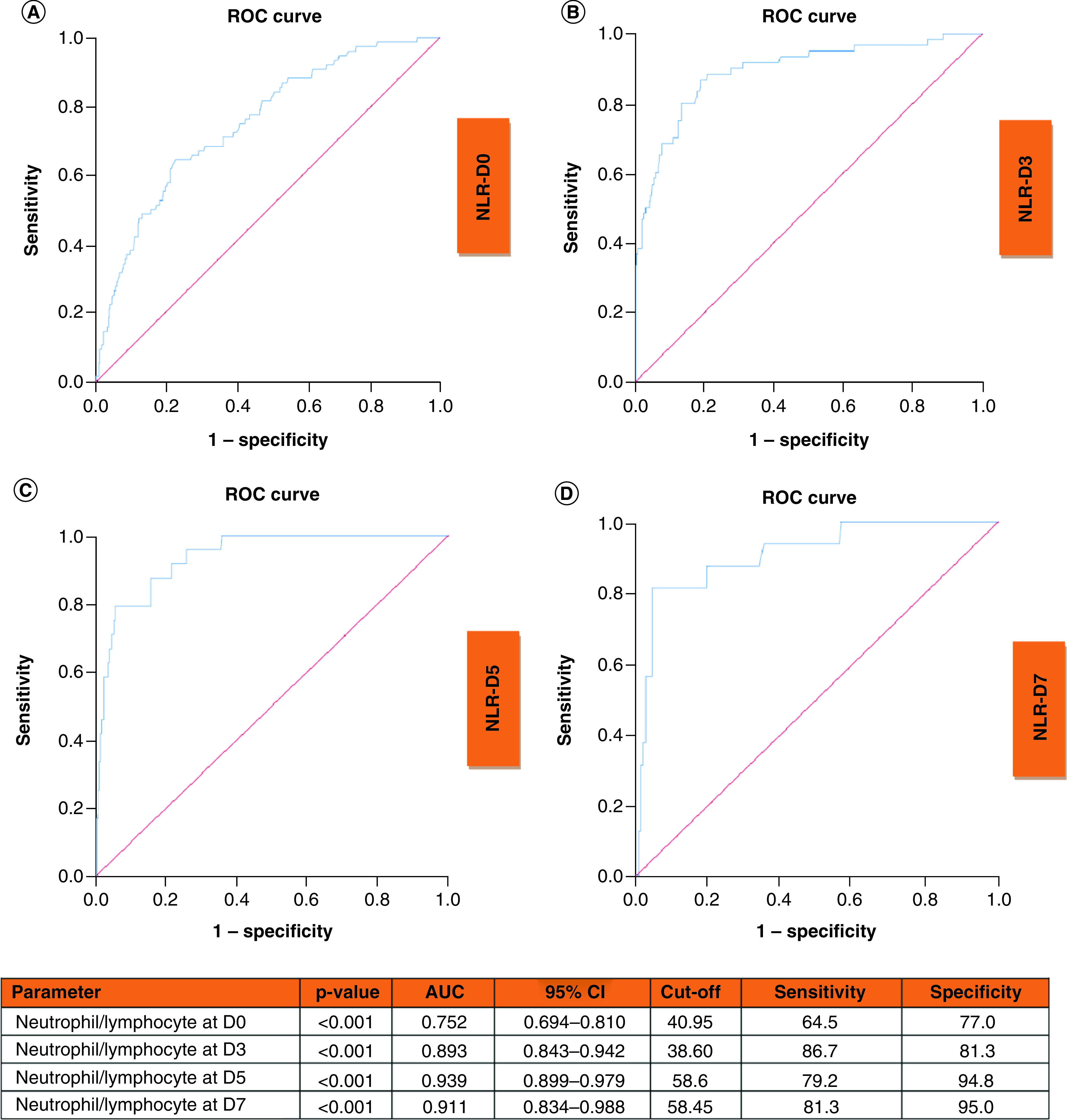
Receiver operating characteristic curves of neutrophil/lymphocyte ratio at D0, D3, D5 and D7 to predict clinical deterioration. Receiver operating characteristic curve and performance value for the best cutoff for: **(A)** Neutrophil/lymphocyte ratio at admission using clinical deterioration. **(B)** Neutrophil/lymphocyte ratio at D3 using clinical deterioration. **(C)** Neutrophil/lymphocyte ratio at D5 using clinical deterioration. **(D)** Neutrophil/lymphocyte ratio at D7 using clinical deterioration. AUC: Area under the ROC curve; D3: Day-3; D5: Day-5; D7: Day-7; NEWS2:National Early Warning Score 2; ROC: Receiver operating characteristic.

ROC curves of NLR ratio at D0, D3, D5 and D7 to predict clinical deterioration are shown in Figure 6. AUC curves at D0, D3, D5 and D7 were 0.752, 0.893, 0.939 and 0.911, respectively (all p < 0.001).

The combined parameter of procalcitonin, NLR, albumin and NEWS2 at D0, D3, D5 and D7 to predict clinical deterioration yielded an increased AUC of 0.838 (95% CI: 0.702–0.883; p < 0.001), 0.947 (95% CI: 0.906–0.988; p < 0.001), 0.989 (95% CI: 0.967–1.000; p < 0.001) and 0.868 (95% CI: 0.647–1.000; p = 0.035), respectively. The sensitivities and specificities of combined use were 70.4 and 88.1 at D0, 95.2 and 82.5 at D3, 83.3 and 98.9 at D5, 66.7 and 97.4% at D7, respectively.

Additionally, we analyzed the association of NEWS2, procalcitonin, albumin and NLR at admission with the end point events at D3, D5 and D7. Albumin was the best predictor for the 3-day end-point. AUC was 0.849 (95% CI: 0.755–0.944), sensitivity of 84.1% and specificity of 72.2% (p < 0.001). Procalcitonin was the best predictor for the 5-day and 7-day end points. AUC was 0.835 (95% CI: 0.748–0.821), with a sensitivity of 79.2 and specificity of 77.1% (p < 0.001) for the 5-day end-point. AUC was 0.849 (95% CI: 0.773–0.924), sensitivity of 79.3 and specificity of 78.2% (p < 0.001).

## Discussion

In this study, we presented a detailed analysis of the NEWS2 score and laboratory parameters in hospitalized patients with COVID-19. Our results showed that NEWS2, procalcitonin, NLR and albumin at D0, D3, D5, and D7 were the best predictors for clinical deterioration (ICU admission or in-hospital death). The combined effect of NEWS2, procalcitonin, NLR and albumin were more valuable to predict clinical deterioration. Procalcitonin had the highest odds ratio (OR) for clinical deterioration at D0, D3, D5 and D7 in univariate analysis. ROC analyses showed that NEWS2 at D7, procalcitonin at D5, albumin at D7 and NLR at D5 had highest AUC values. Additionally, we detected a strong correlation between NEWS2 and laboratory parameters including lymphocyte count, neutrophil count, NLR, PLR, CRP, procalcitonin, ferritin and urea at D0, D3, D5 and D7.

Early and accurate discrimination of need for ICU improves the clinical course of COVID-19 and reduce unnecessary use of ICU beds. There are several published studies on the use of NEWS2 in COVID-19 patients [2–4,7–13]. However, most studies evaluate NEWS2 at admission only [11–13]. In the study of Sze *et al.*, they suggested that NEWS2 score was not a valuable tool to predict clinical deterioration in elderly patients with COVID-19 [14]. However, they reported the results of only 17 elderly patients. Kim *et al.* showed that NEWS2 scores on D0 significantly differed in noncritical and critical patients (2.6 ± 2.6 vs 8.2 ± 3.3; p < 0.001) [9]. In the study of Volff *et al.* the AUC value of NEWS score to predict ICU admission or death was 0.74, in consistent with our result. However, the AUC values of NEWS2 at D3, D5 and D7 were 0.798, 0.833 and 0.842 whereas NEWS2 at D0 was not accurate (AUC <0.750) [7]. Similarly, Sixt *et al.* showed that AUC values of NEWS2 was 0.74 at D0, with a best cutoff of six and was 0.98 at D7, with a best cut off of seven. They reported high sensitivity and specificity at D7 (92 and 97%, respectively) [8].

Due to the physiopathological changes, deterioration in different laboratory parameters occurs while the disease progresses. Therefore, laboratory parameters are commonly used for assessing disease severity. Lagadinou *et al.* found an association between the severity of COVID-19 and the following laboratory parameters NLR, LDH, D-dimers, CRP, fibrinogen and ferritin [15]. In the study of Xu *et al.* procalcitonin, CRP and NLR were valuable predictors for COVID-19 mortality. They showed that the AUC from highest to lowest was combined effect >CRP >procalcitonin >NLR, respectively [16]. Liao *et al.* reported that NLR, thrombocytopenia, prothrombin time and D-dimer were associated with death. They showed that increased NLR (≥9·13) was associated with fivefold increased mortality risk [17]. Similarly, in our study, increased NLR was associated with 1.3-fold at D0, 1.8-fold at D3, 1.8-fold at D5 and 1.3-fold at D7 increased mortality risk in univariate analysis. In a meta-analysis, Elshazli *et al.* demonstrated that higher levels of leukocyte (OR: 5.21), neutrophil (OR: 6.25), D-dimer (OR: 4.19) and prolonged PT (OR: 2.18) was associated with ICU admission. IL-6 (OR: 13.87), CRP (OR: 7.09), D-dimer (OR: 6.36), and neutrophils (OR: 6.25) had the highest ORs for mortality [18]. In a meta-analysis, Lippi *et al.* showed that increased procalcitonin values are associated with a nearly fivefold higher risk for need for ICU or use of mechanical ventilation (OR: 4.76; 95% CI: 2.74–8.29) [19]. Xu *et al.* found that procalcitonin (≥0.10 ng/ml, HR: 12.82), CRP (≥52.14 mg/l, HR: 12.30) and NLR (≥3.59, HR: 8.6) had higher HRs of 12.82, 12.30 and 8.6 for mortality, respectively. Additionally, procalcitonin (≥0.10 ng/ml) and CRP (≥52.14 mg/l), but not NLR exhibited independent increasing risks of mortality, with HRs of 52.68 (95% CI: 1.77–1571.66) and 5.47 (95% CI: 1.04–28.72), respectively [16].

In the study of Shang *et al.* Spearman’s rank correlation analysis revealed that leukocyte, neutrophil, CRP, procalcitonin and LDH were positively correlated and albumin was negatively correlated with mortality in patients with receiving maintenance hemodialysis. Additionally, they showed that CRP had the highest AUC value (0.895) and the values of AUC of neutrophil count, LDH, leukocyte, albumin and procalcitonin were 0.813, 0.758, 0.757, 0.743 and 0.728, respectively [20]. In contrast, we found that procalcitonin was the best predictor for clinical deterioration our study. The optimal cut-off value of procalcitonin at D0, D3, D5 and D7 were 0.065, 0.125, 0.155 and 0.120 ng/ml and the sensitivity and specificity to predict clinical deterioration were 81.1 and 67.9% on D0, 80.6 and 89.3% on D3, 87.5 and 96.7% on D5 and 85.7 and 88.2% on D7, respectively.

Procalcitonin is not well studied for COVID-19 cases. However, some studies suggested that increased procalcitonin levels were found to be associated with the disease severity in patients with COVID-19. A meta-analysis showed that severe patients with COVID-19 had increased procalcitonin levels [18,19]. Similarly, we found that procalcitonin was the best prognostic parameter for the clinical deterioration in our study. Elevated procalcitonin levels could be associated with acute secondary bacterial pneumonia or systemic secondary bacterial infection in patients with COVID-19 due to the production and release into the circulation from procalcitonin-producing extrathyroidal tissues [21]. In our study, despite elevated procalcitonin levels, this elevation was limited (the cut-off values were less than 0.5 ng/ml). In a previous study by Xu *et al.*, they suggested that a limited increase in procalcitonin levels (cut-off value = 0.1 ng/ml) could be associated with increased IFN-γ [16].

Low-serum albumin levels in studies with COVID-19 patients are suggested to be associated with an increased risk of mortality [22–25]. In consistent with other studies, our results confirm that albumin is a valuable predictor for ICU admission or in-hospital death. Albumin is a negative acute phase reactant produced in the liver, and causes downregulation of the expression of angiotensin-converting enzyme-2 receptors, which play a role in the cell entry mechanism of SARS-CoV-2. Liu *et al.* reported that albumin was associated with clinical deterioration and significantly higher in patients with the improvement/stabilization than in those with disease progression (36.62 ± 6.60 vs 41.27 ± 4.55 g/l, p = 0.006) [24]. In the study of Aziz *et al.*, mean albumin at D0 was 3.50 g/dl (CI: 3.26–3.74 g/dl) in the severe group and 4.05 g/dl (CI: 3.82–4.27 g/dl) in the nonsevere group (p < 0.001). They reported that hypoalbuminemia was associated with 12.6-fold increased risk of mortality [25].

An increase in neutrophils and a decrease in lymphocytes have been found in various studies. Some studies have shown that NLR may be an important indicator for the severity of COVID-19 patients. Yan *et al.* showed that NLR was significantly correlated with all-cause in-hospital mortality (OR: 44.351; 95% CI: 4.627–425.088) [26]. The NLR reflects the balance between the innate and adaptive immune systems [26] and increased NLR levels were found to be associated with clinical deterioration in COVID-19 [15–18,26].

This study has several limitations. First, it was retrospectively conducted in a single center. Second, this study had a small sample size and a control group was not included. The generalizability of our results may be limited. Thus, we need new large scale studies providing important information to better understand COVID-19 pandemic. Our study has also several strengths. First, we were able to admit all critically ill patients requiring intensive care to the ICU during the first months of pandemic. This prevents a selection bias. Second, longitudinal evaluation of the association between clinical deterioration and the dynamic changes of laboratory parameters was performed, since we regularly monitored laboratory parameters during the clinical course.

## Conclusion

This study provides a list of several laboratory parameters correlated with NEWS2 and potential predictors for ICU admission or in-hospital death during the clinical course of COVID-19. NEWS2, procalcitonin, NLR and albumin have a high accuracy to predict clinical outcomes/disease progression in hospitalized patients and should be considered in the clinical decision of ICU admission. In conclusion, dynamic monitoring of NEWS2 and laboratory parameters is vital for improving clinical outcomes.

**Summary points**

- The parameters associated with admission intensive care unit (ICU) and in-hospital death at (D0), day-3 (D3), day-5 (D5) and day-7 (D7) were National Early Warning Score 2 (NEWS2), lymphocyte count, neutrophil count, platelet count, neutrophil/lymphocyte ratio (NLR), platelet/lymphocyte ratio (PLR), C-reactive protein (CRP), procalcitonin, D-dimer, troponin, aspartate aminotransferase, urea, lactate dehydrogenase and albumin.
- Laboratory parameters correlated with NEWS2 at D0, D3, D5 and D7 were lymphocyte count, neutrophil count, NLR, PLR, CRP, procalcitonin, ferritin and urea.
- Procalcitonin had the highest odds ratio for clinical deterioration on all days.
- Receiver operating characteristic analyses showed that NEWS2 at D7, procalcitonin at D5, albumin at D7 and NLR at D5 had highest area under the curve values.
- In univariate analysis, among parameters associated with ICU admission or in-hospital death at D0, D3, D5 and D7, best predictors were NEWS2, procalcitonin, NLR and albumin.
- This study provides a list of several laboratory parameters correlated with NEWS2 and potential predictors for ICU admission or in-hospital death during the clinical course of COVID-19.
